# Room-temperature ferroelectricity in CuInP_2_S_6_ ultrathin flakes

**DOI:** 10.1038/ncomms12357

**Published:** 2016-08-11

**Authors:** Fucai Liu, Lu You, Kyle L. Seyler, Xiaobao Li, Peng Yu, Junhao Lin, Xuewen Wang, Jiadong Zhou, Hong Wang, Haiyong He, Sokrates T. Pantelides, Wu Zhou, Pradeep Sharma, Xiaodong Xu, Pulickel M. Ajayan, Junling Wang, Zheng Liu

**Affiliations:** 1 Centre for Programmed Materials, School of Materials Science and Engineering, Nanyang Technological University, Singapore 639798, Singapore; 2 School of Materials Science and Engineering, Nanyang Technological University, Singapore 639798, Singapore; 3 Department of Physics, University of Washington, Seattle, Washington 98195, USA; 4 School of Civil Engineering, Hefei University of Technology, Hefei 230009, China; 5 Department of Physics and Astronomy, Vanderbilt University, Nashville, Tennessee 37235, USA; 6 Materials Science & Technology Division, Oak Ridge National Laboratory, Oak Ridge, Tennessee 37831, USA; 7 Department of Mechanical Engineering, University of Houston, Houston, Texas 77204, USA; 8 Department of Materials Science and NanoEngineering, Rice University, Houston, Texas 77005, USA; 9 NOVITAS, Nanoelectronics Centre of Excellence, School of Electrical and Electronic Engineering, Nanyang Technological University, Singapore 639798, Singapore; 10 CINTRA CNRS/NTU/THALES, UMI 3288, Research Techno Plaza, 50 Nanyang Drive, Border X Block, Level 6, Singapore 637553, Singapore; 12 Present address: National Institute of Advanced Industrial Science and Technology, Tsukuba 305-8565, Japan

## Abstract

Two-dimensional (2D) materials have emerged as promising candidates for various optoelectronic applications based on their diverse electronic properties, ranging from insulating to superconducting. However, cooperative phenomena such as ferroelectricity in the 2D limit have not been well explored. Here, we report room-temperature ferroelectricity in 2D CuInP_2_S_6_ (CIPS) with a transition temperature of ∼320 K. Switchable polarization is observed in thin CIPS of ∼4 nm. To demonstrate the potential of this 2D ferroelectric material, we prepare a van der Waals (vdW) ferroelectric diode formed by CIPS/Si heterostructure, which shows good memory behaviour with on/off ratio of ∼100. The addition of ferroelectricity to the 2D family opens up possibilities for numerous novel applications, including sensors, actuators, non-volatile memory devices, and various vdW heterostructures based on 2D ferroelectricity.

## Abstract (web summary)

Two dimensional materials are promising for electronic applications, which await the exploration of cooperative phenomena. Here, Liu *et al.* report switchable ferroelectric polarization in thin CuInP_2_S_6_ film at room temperature, demonstrating good memory behaviour with on/off ratio of ∼100 based on two-dimensional ferroelectricity.

## Graphical abstract

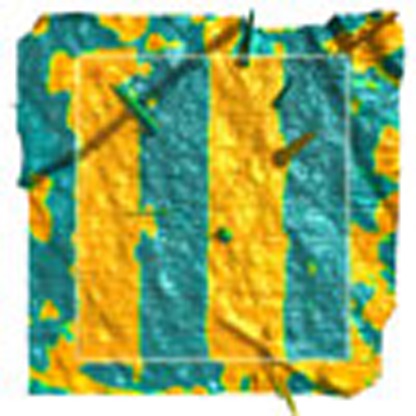

Ferroelectricity is a collective property of certain materials in which macroscopic polarization arises from spontaneous ordering of electric dipoles and can be switched by external electric field. Most technologically important ferroelectrics are perovskite oxides with strong covalent/ionic bonds, such as PbTiO_3_ and BaTiO_3_, which have been widely applied in electronic and optoelectronic devices^1^,^2^,^3^. Due to the three-dimensional nature of the ferroelectric oxide lattices, epitaxial growth of high-quality films requires the careful selection of substrates with small lattice mismatch^4^. This severely limits the possible materials that can be utilized in ferroelectric heterostructure devices. In addition, prevalent dangling bonds and defects at the interface drastically deteriorate the electronic coupling between ferroelectric and graphene like two-dimensional (2D) materials^5^, due to the complex interface reconstruction and defect chemistry^6^. Studying weakly bonded non-oxide ferroelectric compounds is thus both fundamentally and practically rewarding. Meanwhile, the groundbreaking work on graphene has triggered an intense search for other 2D materials with intriguing physical properties^7^,^8^,^9^. However, ferroelectricity has so far remained elusive to the 2D material library. Currently the reported critical thickness for ferroelectricity in layered materials is relatively large (above 50 nm thick)^10^, far from the ultrathin limit.

Here, we report the experimental observation of switchable polarization in CuInP_2_S_6_ (CIPS) films down to 4 nm at room temperature. Second-harmonic generation (SHG) measurements show the transition from ferroelectric to paraelectric accompanies the structural change from inversion asymmetric to symmetric. Finally, we demonstrate a non-volatile memory device with on/off ratio of ∼100 in a CIPS/Si ferroelectric diode.

## Results

### Characterization of CuInP_2_S_6_

**Figure 1:**
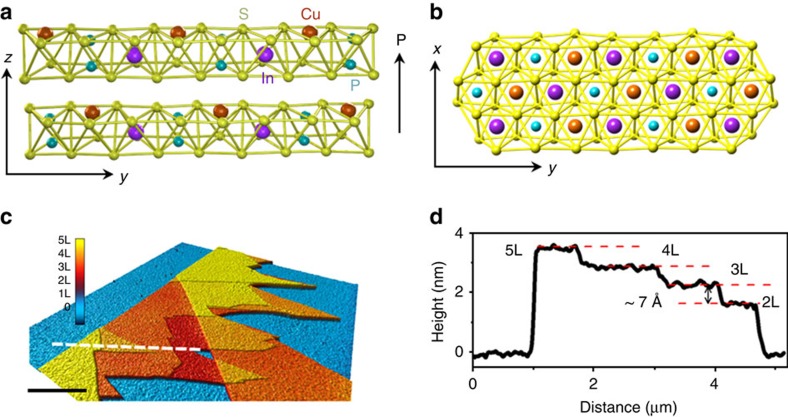
Crystal structure and characterization of CIPS. (**a**,**b**) The side view (**a**) and side view (**b**) for the crystal structure of CIPS with vdW gap between the layers. Within a layer, the Cu, In and P–P form separate triangular networks. The polarization direction is indicated in by the arrow. (**b**) The ferroelectric hysteresis loop of a 4-μm-thick CIPS flake. (**c**) AFM image of the CIPS flakes with different thicknesses. Scale bar, 2 μm. (**d**) The height profile along the line shown in **c**. Clear step height of 0.7 nm corresponding to single layer thickness of CIPS can be observed. L, Layers.

CIPS is one of the few layered compounds which exhibits room-temperature ferroelectricity^11^. The atomic structure of CIPS contains of a sulfur framework with the octahedral voids filled by the Cu, In and P–P triangular patterns. Bulk crystals are composed of vertically stacked, weakly interacting layers packed by van der Waals interactions (Fig. 1a,b)^12^. Owing to the site exchange between Cu and P–P pair from one layer to another, a complete unit cell consists of two adjacent monolayers to fully describe the material's symmetry. It is a collinear two-sublattice ferrielectric system with *T*_c_ of about 315 K (ref. 11). When the temperature drops below *T*_c_, due to the off-centre ordering in the Cu sublattice and the displacement of cations from the centrosymmetric positions in the In sublattice (symmetry changes from C_2/c_ to C_c_), spontaneous polarization emerges in the ferrielectric phase with polar axis normal to the layer plane^13^. For simplicity, CIPS will be referred as ferroelectric because ferrielectric materials exhibit the same macroscopic properties as ferroelectrics: namely, a spontaneous and switchable net electric polarization. To verify the ferroelectricity of the bulk sample, polarization versus out-of-plane electric field curve was measured on a 4-μm-thick CIPS flake using a commercial ferroelectric analyzer, where the clear hysteresis loop is direct evidence of ferroelectricity^13^. Details of the ferroelectric and dielectric measurements as well as the temperature dependence are shown in Supplementary Note 1 and Supplementary Figs 1 and 2.

The weak interlayer vdW interaction in CIPS allows us to exfoliate ultrathin flakes from a single crystal and study the ferroelectric properties with reduced dimension. Flakes with different thicknesses were mechanically exfoliated on heavily doped Si substrate with or without SiO_2_, depending on the purpose of the measurement. They were identified by optical contrast in a microscope and the thickness was subsequently measured using an atomic force microscopy (AFM). Figure 1c shows a typical AFM image of CIPS flakes with different thicknesses on a Si substrate covered with 285 nm SiO_2_. In the height profile (Fig. 1d) along the line in Fig. 1c, flakes from two to five layers thick and clear monolayer steps are observed. The atomic structure and high quality of the CIPS crystal is also confirmed by high-resolution scanning transmission electron microscopy (STEM) imaging (Supplementary Fig. 3) and Raman spectroscopy (Supplementary Fig. 4).

### Piezoresponse force microscopy measurement

**Figure 2:**
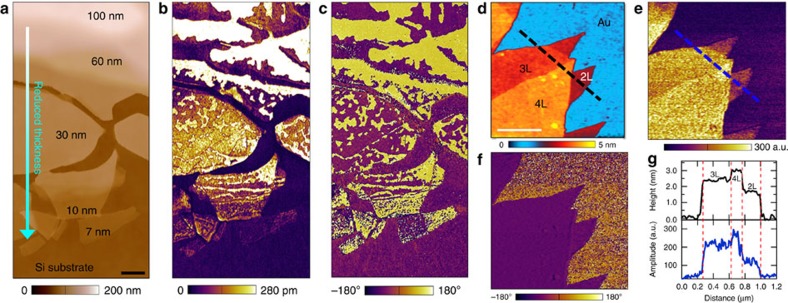
AFM and piezoresponse images of CIPS with different thicknesses. (**a**–**c**) AFM topography (**a**) PFM amplitude (**b**) and PFM phase (**c**) for CIPS flakes ranging from 100 to 7 nm thick, on doped Si substrate. Scale bar in **a**, 1 μm. (**d**,**e**) AFM topography (**d**) PFM amplitude (**e**) and phase (**f**) of 2–4 layer thick CIPS on Au coated SiO_2_/Si substrate. Scale bar in **d**, 500 nm. (**g**) the height (black) and PFM amplitude (blue) profile along the lines shown in **d** and **e**, respectively. L, Layers.

To verify the thin-film ferroelectricity, CIPS flakes with various thicknesses were investigated using piezoresponse force microscopy (PFM) under both resonant and non-resonant modes (see Methods). The PFM amplitude reflects the absolute magnitude of the local piezoelectric response, while the phase indicates the polarization direction in each individual domain^14^. Figure 2a–c shows the typical domain evolution of CIPS flakes with thickness ranging from 100 nm down to sub-10 nm. With reduced flake thickness (Fig. 2a), the PFM amplitude signal also decreases (Fig. 2b). Similar behaviour is commonly observed in conventional ferroelectric films due to the enhanced depolarization effect in thinner films^15^ as well as the nonuniform electric field of the AFM tip^16^. However, the amplitude signal above the background persists down to the lowest thickness (7 nm). The PFM phase image (Fig. 2c) is characterized by two-colour tones with a contrast of 180°, corresponding to the two opposite polarization directions perpendicular to the layer surface. To confirm the polarization is confined in vertical direction, thickness-dependent vector (vertical and lateral) PFM is conducted on flakes with thickness ranging from sub-10 to 100 nm (Supplementary Figs 5 and 6 and Supplementary Note 2). Persistent noise-level in-plane piezoresponse signal suggests negligible effect of possible residual strain on the polarization orientation^17^, thanks to the quasi-freestanding nature of the specimen. The domain patterns evolve from fractal in thinner flakes towards dendrite-like in thicker ones, together with an increase in domain size. The piezoresponse amplitude reduces with the layer thickness, consistent with a finite depolarization effect commonly found in ferroelectric thin films^18^,^19^. These observations imply that CIPS ultrathin flakes remain ferroelectric down to a few nanometre thick. Piezoelectric response was also obtained from a bilayer (∼1.6 nm) CIPS flake (Fig. 2d–g), benefiting from the absence of surface/interface reconstructions in 2D materials. To further examine the ferroelectric polarization in ultrathin CIPS, we calculated the structure and polarization of CIPS by the density functional theory (DFT) calculation, and found that the ferrielectric phase can be stabilized in bilayer CIPS (see Methods and Supplementary Note 3 and Supplementary Fig. 7 for details).

### Ferroelectric switching

**Figure 3:**
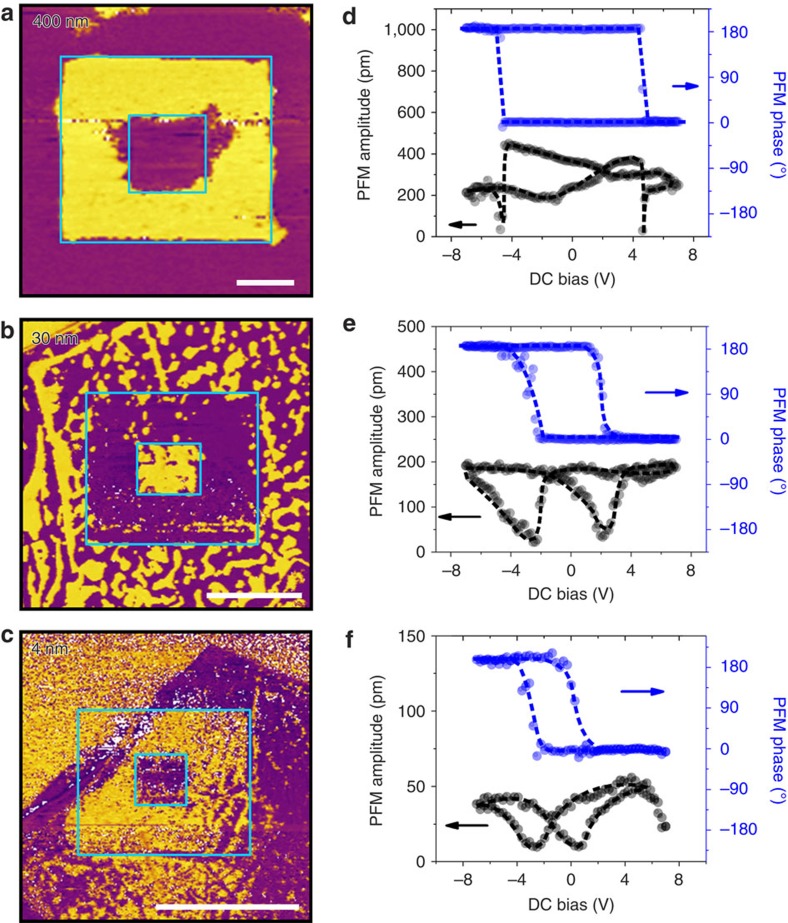
Ferroelectric polarization switching by PFM for CIPS flakes with different thicknesses. (**a**–**c**) The PFM phase images for 400 nm (**a**) 30 nm (**b**) and 4 nm (**c**) thick CIPS flakes with written box-in-box patterns with reverse DC bias. Scale bar, 1 μm. (**d**–**f**) The corresponding PFM amplitude (black) and phase (blue) hysteresis loops during the switching process for 400 nm (**d**) 30 nm (**e**) and 4 nm (**f**) thick CIPS flakes.

By definition, a ferroelectric material should possess spontaneous polarization that is switchable. Although hysteresis loops have been obtained in thick CIPS flakes, it does not warrant switchable polarization in thinner samples, as conventional ultrathin ferroelectric films are notorious for their deteriorated ferroelectric performance owing to the depolarization effect^18^, interface/surface polarization pinning^20^, or, more generally, the ‘dead layer' effect^21^. Hence, we carried out local switching tests by applying a bias between the conductive PFM tip and the heavily doped Si substrate. Figure 3a–c displays the PFM phase images of 400, 30 and 4 nm thick CIPS flakes after writing box-in-box patterns with reversed DC bias in the centre (see Supplementary Figs 8–12 and Supplementary Note 4 for detailed results). Clear reversal of phase contrast confirms the switching of polarization in CIPS down to ∼4 nm. Meanwhile, there are no obvious changes in the corresponding surface morphologies as previously reported^10^. Furthermore, the CIPS flakes exhibit superior ferroelectric retention despite the relatively low ferroelectric Curie temperature (Supplementary Fig. 13). The written domain patterns are still discernible after several weeks in ambient condition. These observations clearly rule out the possible contribution of PFM signal from the electrochemical phenomena^22^,^23^,^24^. The corresponding switching spectroscopic loops were also recorded under resonance-enhanced PFM mode by applying an AC electric field superimposing on a DC triangle saw-tooth waveform (Supplementary Fig. 14). The well-defined butterfly loops of the PFM amplitude signals and the distinct 180° switching of the phase signals further corroborate the robust ferroelectric polarization in CIPS ultrathin flakes (Fig. 3d–f). Furthermore, the longitudinal piezoelectric coefficient of the CIPS flakes were quantitatively determined using off-resonant PFM (Supplementary Note 5 and Supplementary Figs 15 and 16).

### Second-harmonic generation measurement

**Figure 4:**
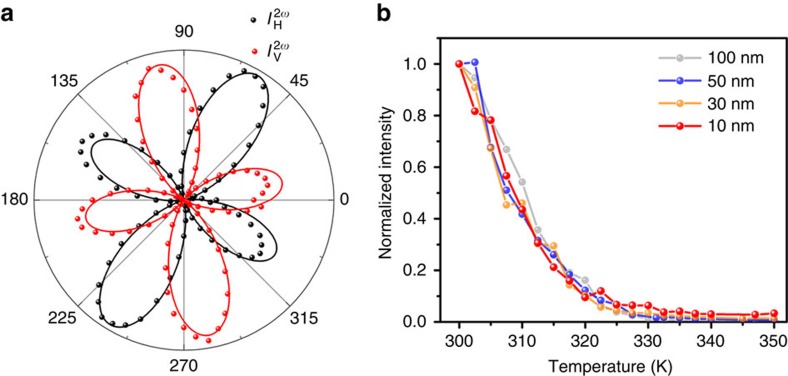
Thicknesses-dependent second-harmonic generation (SHG). (**a**) Polar plots of the SHG intensity in H and V directions (laboratory coordinates) as a function of the excitation laser linear polarization for a 100 nm thick CIPS flake. (**b**) Temperature dependence of the SHG intensity for CIPS flakes with thickness of 100, 50, 30 and 10 nm, respectively. The SHG intensity of each thickness is normalized to its intensity at 300 K.

To probe the structural phase change accompanying the ferroelectric to paraelectric transition, we utilized SHG microscopy, which is a sensitive probe of broken inversion symmetry and an excellent tool for the investigation of ferroelectric order^25^,^26^. We first explored the structural symmetry through polarization-resolved SHG at 300 K under normal incidence excitation (see Methods and Supplementary Information for details of the measurement). Figure 4a illustrates the SHG intensity dependence on excitation polarization for fixed detection along the horizontal (H) or vertical (V) direction (laboratory coordinates), which are fit well (solid lines) using the allowed in-plane second-order susceptibility elements for point group m (ref. 27; see Supplementary Note 6 and Supplementary Fig. 17). The nonzero SHG directly reveals the broken inversion symmetry that generates the ferroelectricity. This is clearly seen in the approximate sixfold rotational symmetry of the co-linearly polarized SHG (Supplementary Fig. 5), which reflects hexagonal ordering of the displaced Cu and In sublattices in the ferroelectric phase. We further investigated the SHG intensity as a function of temperature for CIPS flakes with thicknesses from 100 to ∼10 nm. Figure 4b shows the normalized intensity plotted as a function of temperature. All flakes follow the same trend: below *T*_c_, there is significant SHG signal, but as the temperature increases, the SHG intensity decreases gradually, and almost vanishes at high temperature. This is a strong indication of the ferroelectric to paraelectric phase transition around *T*_*c*_, which involves a structural change from noncentrosymmetric (m) to centrosymmetric (2/m).

### Ferroelectric diode based on CIPS/Si heterostructure

**Figure 5:**
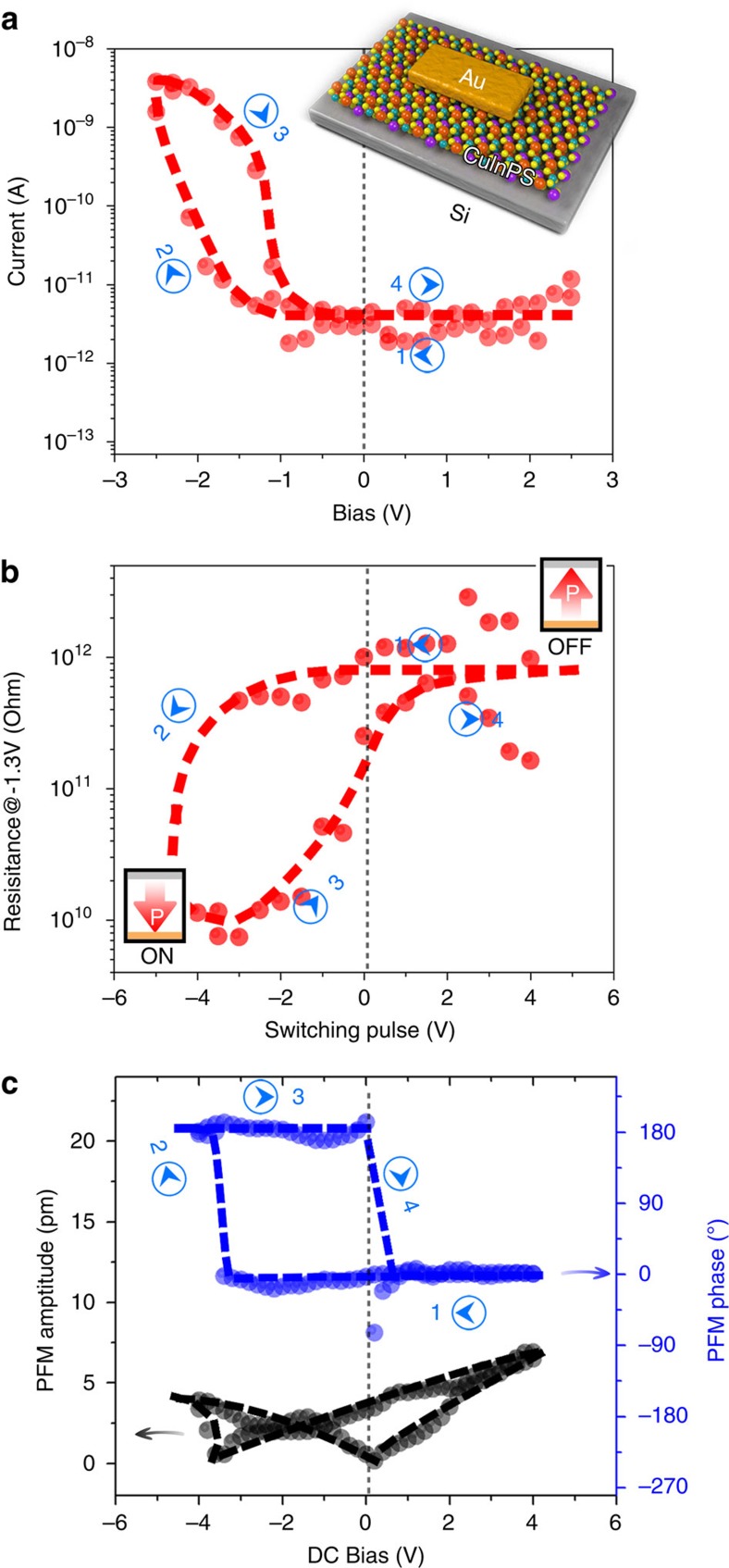
Electric characterization of the vdW CIPS/Si diode. (**a**) The *I*–*V* curves from the typical vdW CIPS/Si diode with 30 nm thick CIPS, by sweeping the bias from 2.5 to −2.5 V, and then back to 2.5 V. Inset is the schematic of the device. (**b**) Resistance-switching voltage hysteresis loop of the diode measured at a bias voltage of −1.3 V. The schematic representations of the ON and OFF states with respect to the polarization direction are shown in the bottom-left and top-right insets, respectively. (**c**) Out-of-plane PFM amplitude (black) and phase (blue) measurements on the same diode device shown in **a**.

All the above evidence unambiguously establishes the existence of ferroelectric order in 2D layers of CIPS, making it a promising non-volatile element in vdW heterostructures. We test its applications in a prototype ferroelectric diode (inset of Fig. 5a). The vdW heterojunction was fabricated by exfoliating the CIPS flakes (30 nm) on to a Si substrate and followed by patterning the top electrodes (see Methods). Electrical contact to the top electrode was made using an AFM conductive tip. Figure 5a shows the current change by sweeping the bias from 2.5 V to −2.5 V, and back to 2.5 V. Figure 5b shows the resistance calculated at a bias of −1.3 V while sweeping the switching pulse. A clear large hysteresis and resistive switching are observed. The ON and OFF states, which correspond to the low- and high-resistance states, respectively, can be assigned. The resistive switching and resulting memory effect is due to the polarization switching of the ferroelectric CIPS layer, as evidenced from the piezoelectric switching measurement of the same device (Fig. 5c). The coercive voltage coincides minima in the amplitude loop as well as the switching bias in the phase signal coincides with the bias, at which resistive switching takes place. This strongly suggests that the ferroelectric polarization reversal is the origin of the resistive switching in the vdW diode. The on/off ratio of about 100 is comparable to that observed in tunnel junctions based on conventional ferroelectric oxide^28^. These results, although obtained on unoptimized devices, constitute a proof of concept for novel non-volatile memories based on ferroelectric 2D materials.

## Discussion

In summary, we have unambiguously established room-temperature ferroelectricity in ultrathin CIPS flakes of ∼4 nm thick as well as piezoelectric response in bilayer CIPS. A simple vdW CIPS/Si ferroelectric diode exhibits non-volatile memory behaviour with on/off ratio of ∼100, exhibiting the capability of integration with well-established Si-based platforms. Our discovery greatly enriches the functionalities of the 2D material family and opens new possibilities for novel devices based on vdW heterostructures.

## Methods

### Sample preparation and characterization

High-quality single crystals of CIPS were synthesized by solid state reaction as previously reported^12^. The thin flakes were obtained by mechanical exfoliation from synthetic bulk crystals onto heavily doped silicon substrates with or without a 285 nm SiO_2_ layer on top. The thickness of the flakes was identified from their optical contrast and AFM. Raman spectrum was carried out using a confocal Raman system (WITec) with the 532 nm laser excitation. SHG measurements were performed in reflection geometry with 100 fs pulses at 786 nm and a repetition rate of 76 MHz, which were focused to a spot size of ∼1 μm by a 40 × 0.6 NA objective lens (Olympus). TEM sample was prepared by dropcasting the solution, which contains exfoliated thin flakes after sonication of the thick CIPS crystal, onto a lacy carbon TEM grid. Z-contrast STEM imaging was performed on a Nion UltraSTEM-100, equipped with a fifth order aberration corrector, operated at 60 kV. The convergence angle is set to be ∼30 mrad. All Z-contrast STEM images were acquired from the ∼86–200 mrad range.

### PFM and ferroelectric polarization measurement

PFM measurement was carried out on a commercial atomic force microscope (Asylum Research MFP-3D) under both resonance-enhanced and off-resonance modes. In resonance-enhanced mode, a soft tip with a spring constant of ∼2 N m^−1^ was driven with an ac voltage (Vac=0.5–1 V) under the tip-sample contact resonant frequency (∼300 kHz). In off-resonance mode, a stiff tip with a spring constant of ∼40 N m^−1^ was driven at 10 kHz. The inverse optical lever sensitivity (InvOLS, nm/V) was calibrated beforehand to obtain quantitative piezoelectric displacement data. Vector PFM was performed by imaging both the out-of-plane and in-plane PFM at different azimuth angles between the sample and AFM cantilever. Ferroelectric polarization measurements were carried out using a commercial ferroelectric tester (Radiant Technologies) and a pulse generator (Keithley 3,401). Dielectric permittivity was characterized using a commercial LCR metre (Agilent E4980A).

### Device fabrication and measurement

The ferroelectric diode was fabricated by exfoliating the thin flakes of CIPS onto heavily doped silicon substrates. The top electrodes are defined using standard photolithography process followed by thermal evaporation of the Ti/Au (1 nm/10 nm) metal, and lift-off process. Electrical measurements were performed using a commercial AFM (Asylum Research MFP-3D) integrated with a pA metre/direct current (d.c.) voltage source (Hewlett Package 4140B).

### Computational method

The quantum calculations are based on the density functional theory (DFT) as implemented in the Quantum-Espresso computational package^29^ (http://www.quantum-espresso.org/). The PAW pseudopotentials with PBE exchange correlation functional from Quantum-Espresso pseudopotential database are used for each element in CIPS (http://www.quantum-espresso.org/pseudopotentials/). The 4 × 8 × 1 *k*-point grid by Monkhorst-Pack scheme was selected for the calculations. The energy cutoff for wave function and charge density are set as 50 Ry and 400 Ry respectively. A vacuum region of 20 Å is set in the direction perpendicular to the layer to avoid the interaction between the periodic images. The polarization calculation is performed using Berry-phase method^30^ embedded in the Quantum-Espresso package. In this method, the total polarization includes two contributions: ionic and electronic. More *k*-point (for example, 9) has been used in the polarization calculation direction. All the calculations are done at 0 K.

### Data availability

The data that support the findings of this study are available from the corresponding author upon request.

## Additional information

**How to cite this article:** Liu, F. *et al.* Room-temperature ferroelectricity in CuInP_2_S_6_ ultrathin flakes. *Nat. Commun.* 7:12357 doi: 10.1038/ncomms12357 (2016).

## Supplementary Material

Supplementary Information Supplementary Figures 1-17, Supplementary Notes 1-6 and Supplementary References
