# Supplementary material for: Room-temperature ferroelectricity in CuInP2S6 ultrathin flakes
Source: Nat Commun. 2016 Aug 11;7:12357. doi: 10.1038/ncomms12357 (PMC4987531; doi:10.1038/ncomms12357)
Supplement: Supplementary Information — Supplementary Figures 1-17, Supplementary Notes 1-6 and Supplementary References [file ncomms12357-s1.pdf]

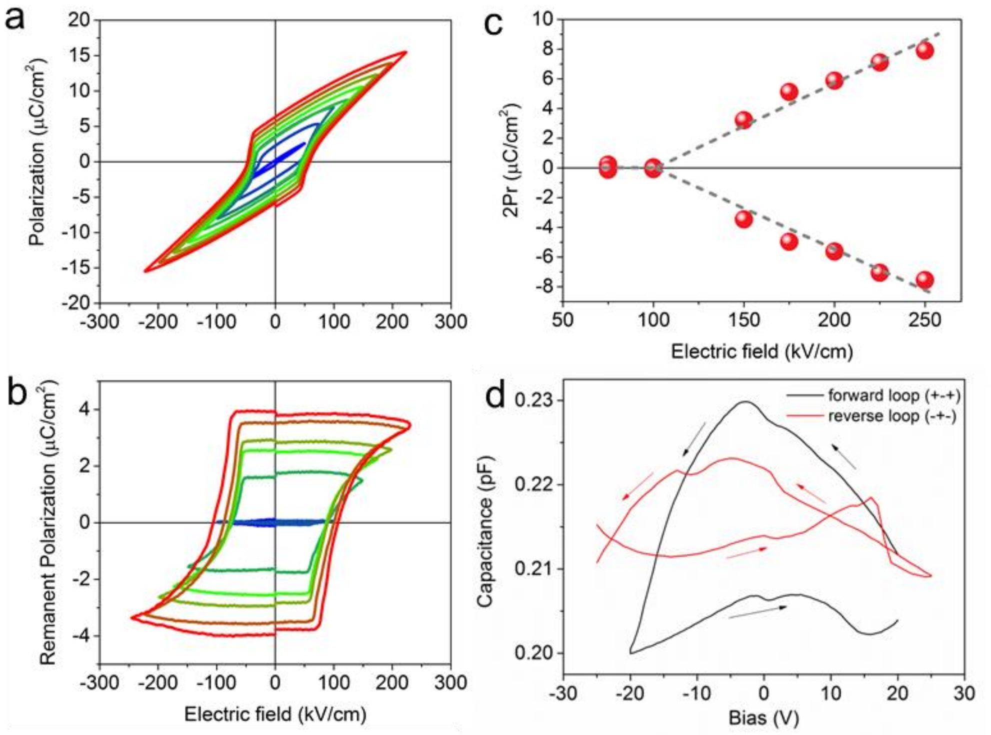

Supplementary Figure 1 | Routine ferroelectric characterizations of a 4-μm-thick CIPS flake. a, Standard ferroelectric polarization-electric field (P-E) hysteresis loops of a 4-μm-thick CIPS flake at 100 Hz. Different trance represents different highest electric field applied during each scan. b, Remnant polarization hysteresis loops of the same flake after subtracting the unswitchable components of the measured polarization. c, Positive-up-negative-down (PUND) measurement of the switchable polarization versus electric field. d, Capacitance-voltage (C-V) hysteresis curves of the same flake under forward (black line) and reverse (red line) DC voltage sweeping. The AC bias is driven at 0.1 V and 100 kHz. The DC sweeping rate is around 0.1 Hz.

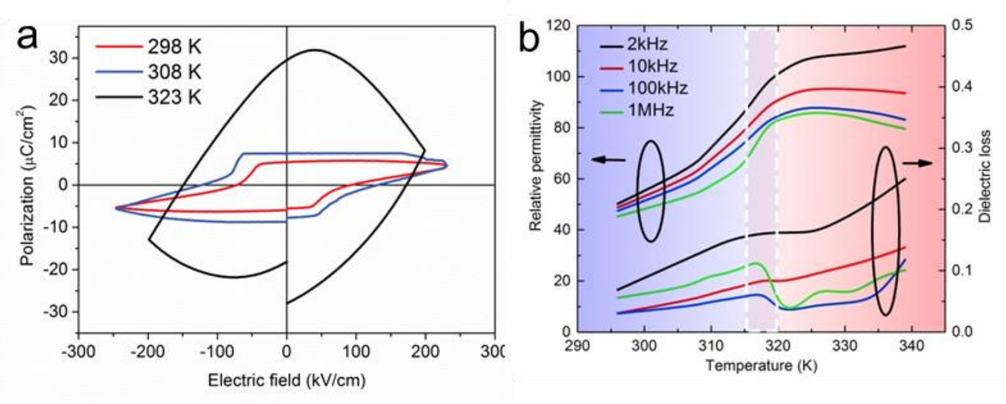

Supplementary Figure 2 | Temperature dependence of the ferroelectric characteristics. a, The ferroelectric hysteresis loop of a 4-μm-thick CIPS flake under temperature of 298 K (black line), 308 K (red line), and 323 K (blue line), respectively. b, Temperature-dependent dielectric permittivity of a 200-nm-thick CIPS flake along the polar axis under different frequencies.

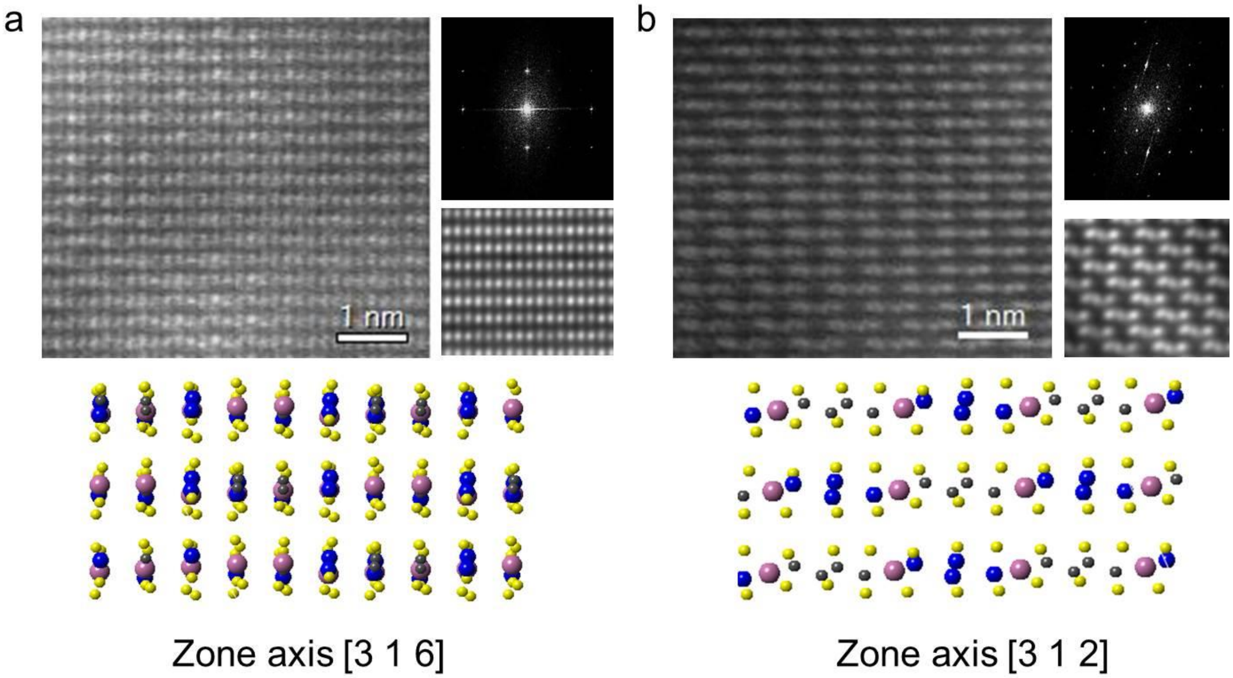

Supplementary Figure 3 | STEM characterizations of CIPS crystal along different zone axis. a-b, The Z-contrast STEM image, corresponding fast Fourier transform (FFT) pattern and projected atomic models along the zone axis [316] (a) and [312] (b), respectively.

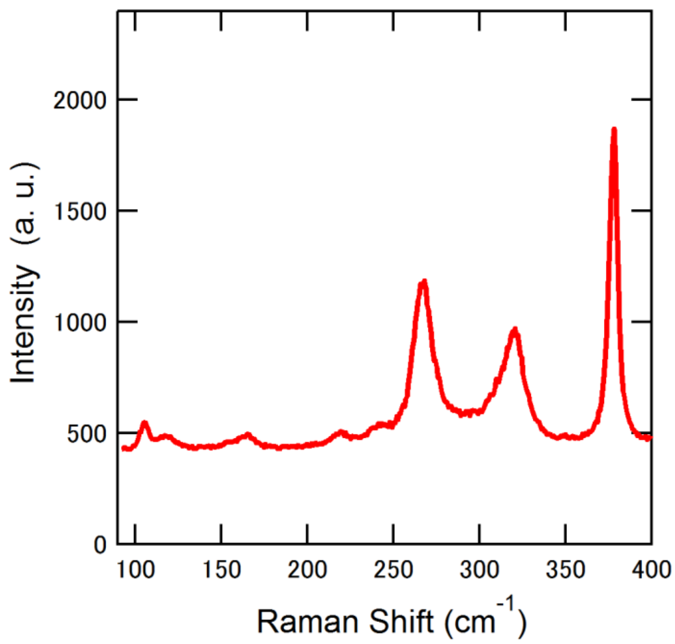

Supplementary Figure 4 | Raman spectrum of CIPS single crystal. Three dominant peaks are observed in the range from 100 cm^{-1} to 450 cm^{-1}, which are assigned to the vibrational modes of the $(P_2S_6)^{4-}$ anions and the cation sites, respectively. The peaks are consistent with the reported Raman spectrum of CIPS, indicating same crystal symmetry of our sample with reported one.

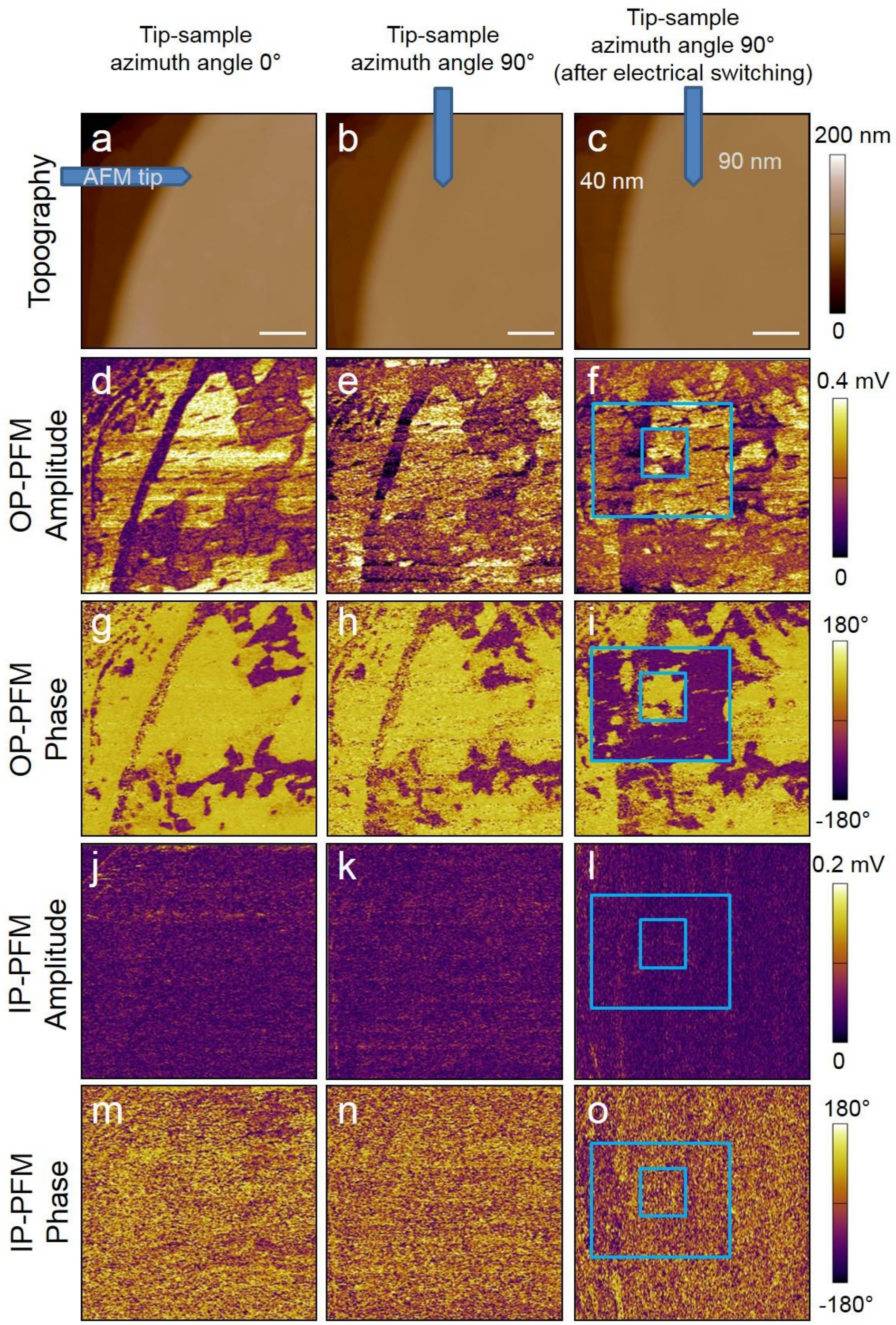

Supplementary Figure 5 | Vertical (out-of-plane, OP) and lateral (in-plane, IP) PFM study of CIPS. a-c, Topography; d-f, OP-PFM amplitude; g-i, OP-PFM phase; j-l, IP-PFM amplitude and m-o, IP-PFM phase images of 20-100 nm thick CIPS flakes with tip-sample azimuth angle at 0 °, 90 °and after electrical switching, respectively. Scale bar, 1 μm.

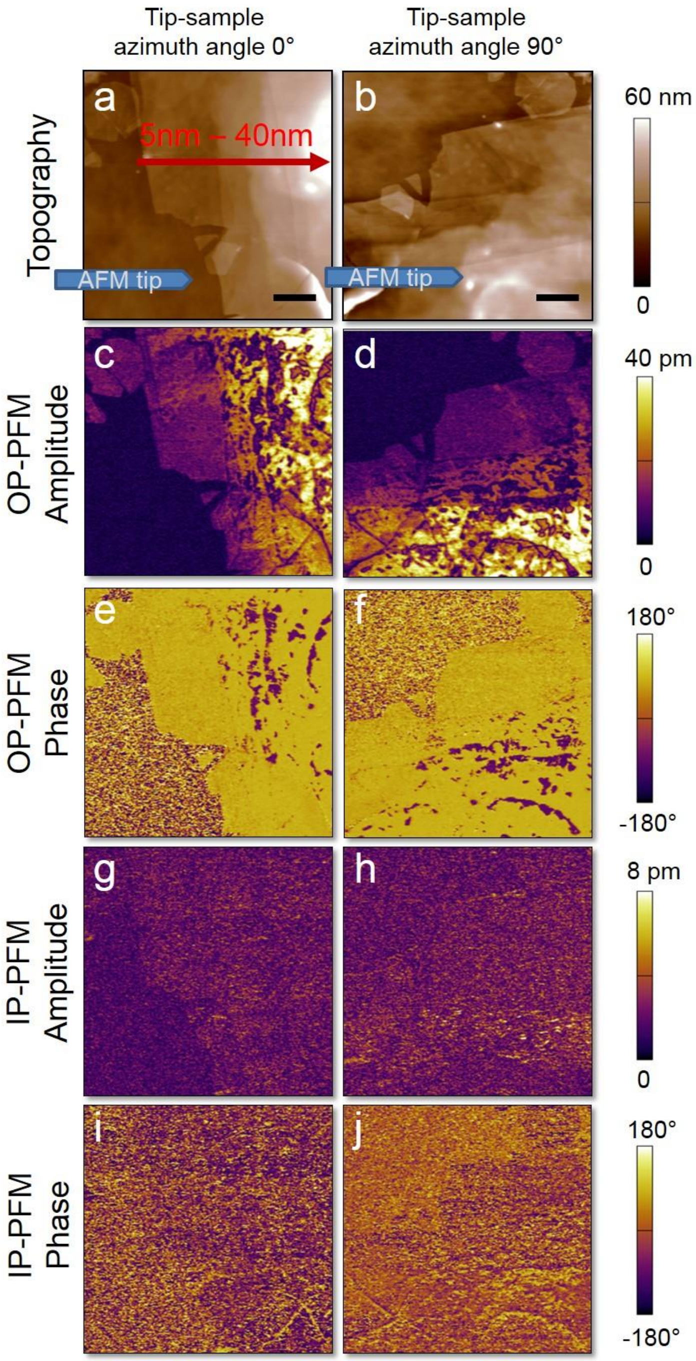

Supplementary Figure 6 | Vertical (out-of-plane, OP) and lateral (in-plane, IP) PFM study of ultrathin CIPS. a-b, Topography; c-d, OP-PFM amplitude; e-f, OP-PFM phase; g-h, IP-PFM amplitude and i-j, IP-PFM phase images of 5-40 nm thick CIPS flakes with tip-sample azimuth angle at 0 ° and 90 °, respectively.

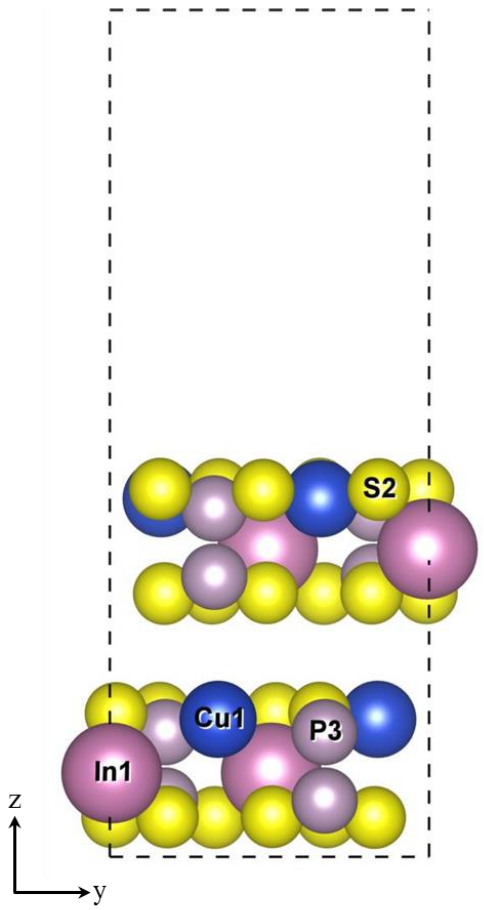

Supplementary Figure 7 | Supercell used in the calculation. 15 Angstrom vacuum is inserted in z direction. The Cu are on the upper-layer site in each layer. Each type of atom is shown in the figure.

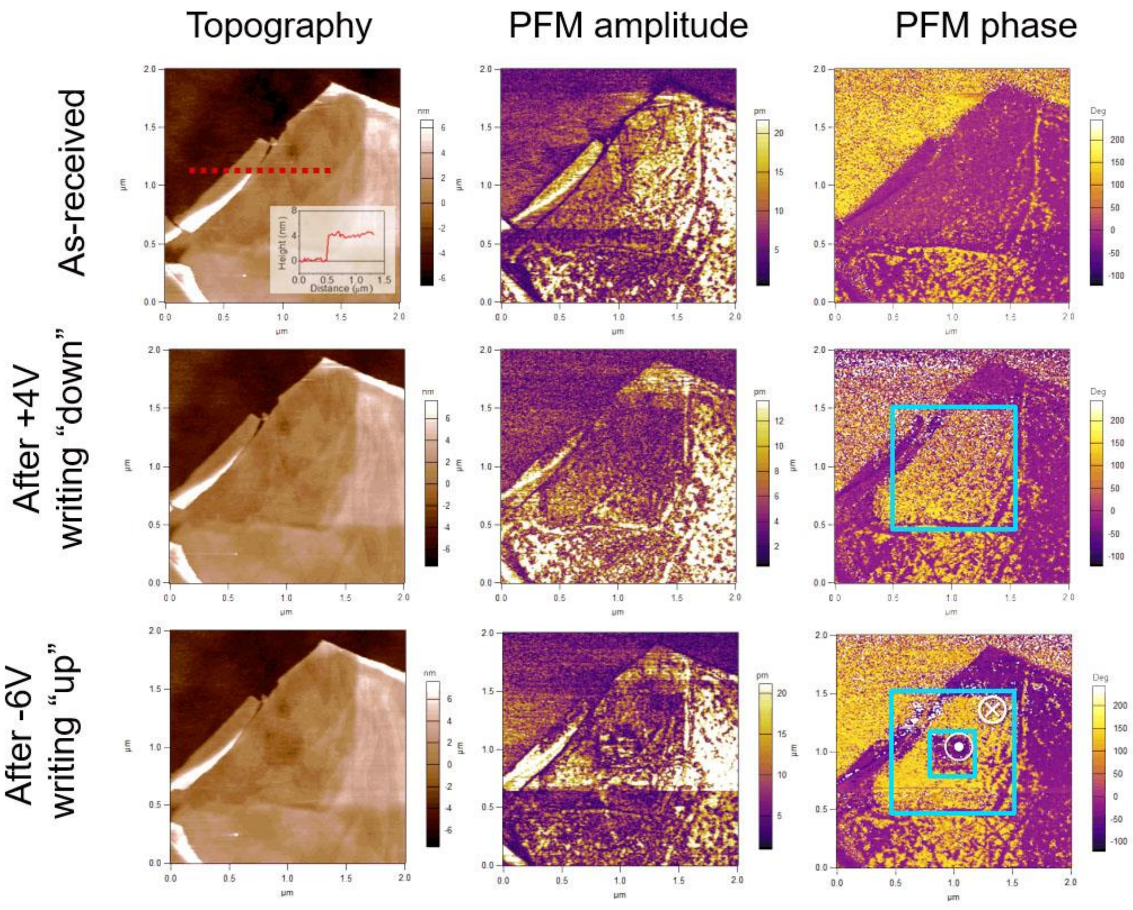

Supplementary Figure 8 | PFM measurement and polarization switching of CIPS with a thickness of 4 nm. The topography, PFM amplitude, PFM phase for the as received, after +4 V, and -6 V bias writing, respectively.

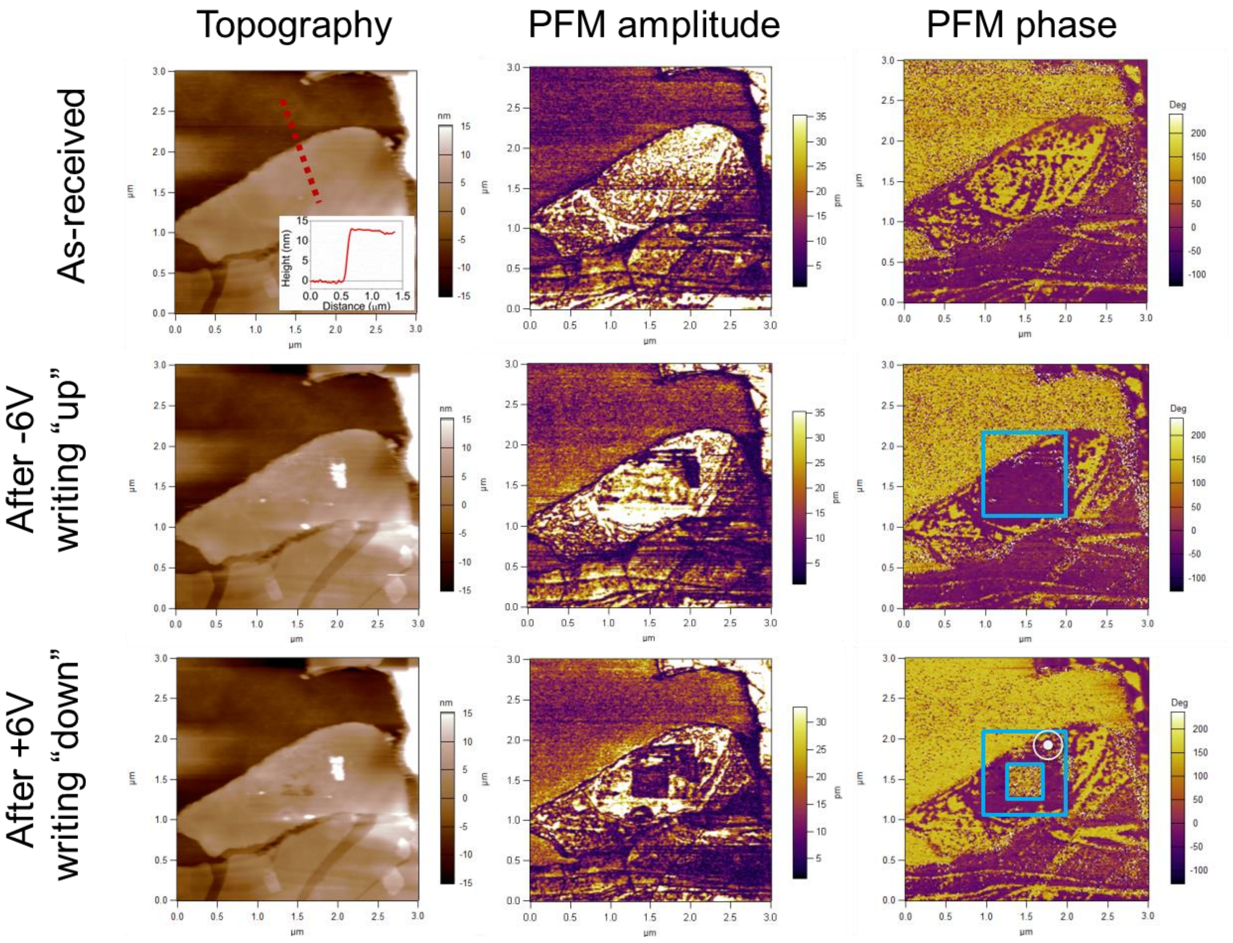

Supplementary Figure 9 | PFM measurement and polarization switching of CIPS with a thickness of 12 nm. The topography, PFM amplitude, PFM phase for the as received, after -6 V, and +6 V bias writing, respectively.

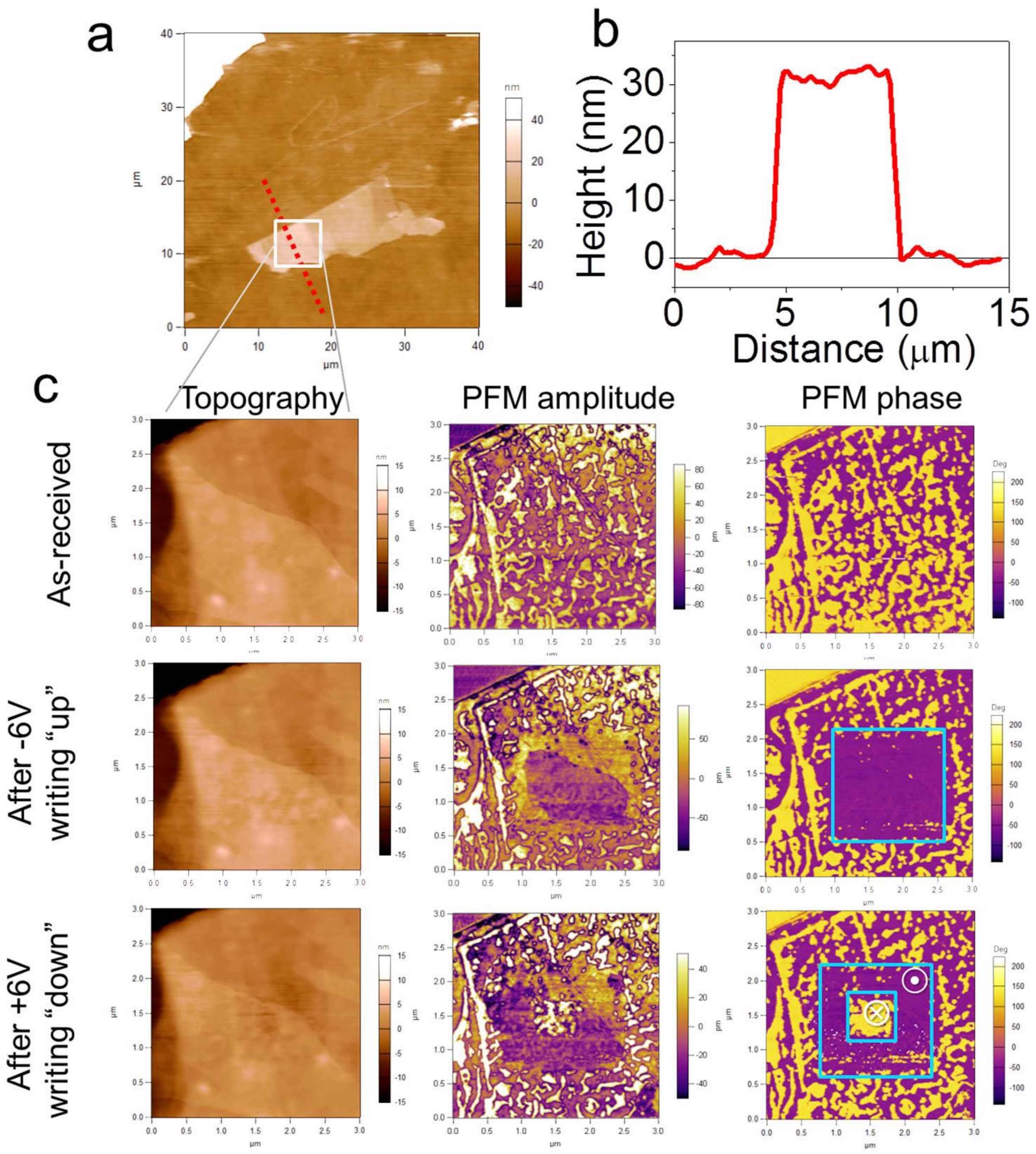

Supplementary Figure 10 | PFM measurement and polarization switching of CIPS with a thickness of 30 nm. a, The topography of the a 30-nm thick CIPS flake. b, The height profile along the dashed line in (a). c, The topography, PFM amplitude, PFM phase for the as received, after -6 V and +6 V bias writing, respectively.

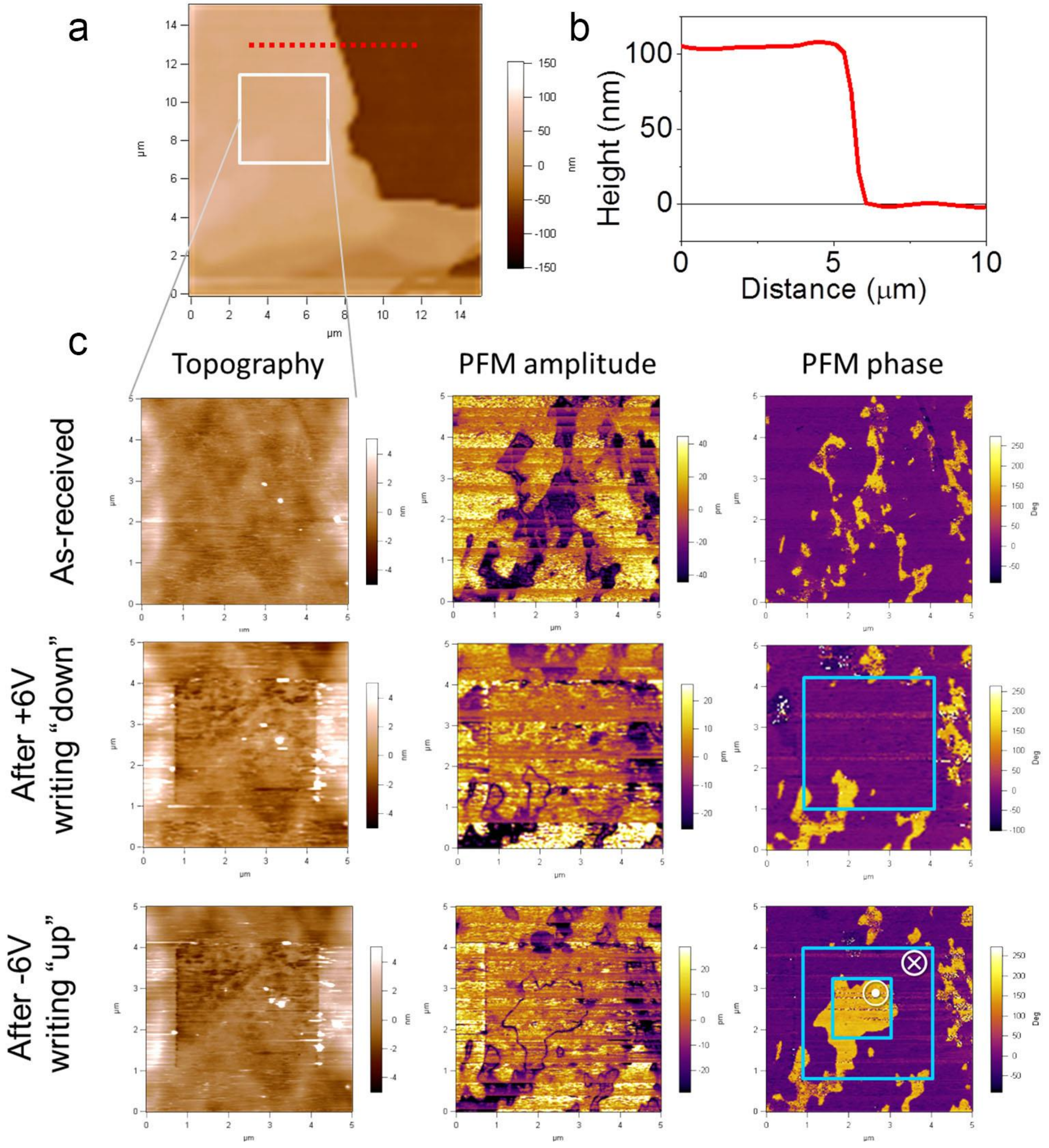

Supplementary Figure 11 | PFM measurement and polarization switching of CIPS with a thickness of 100 nm. a, The topography of the a 100-nm thick CIPS flake. b, The height profile along the dashed line in (a). c, The topography, PFM amplitude, PFM phase for the as received, after -6 V and +6 V bias writing, respectively.

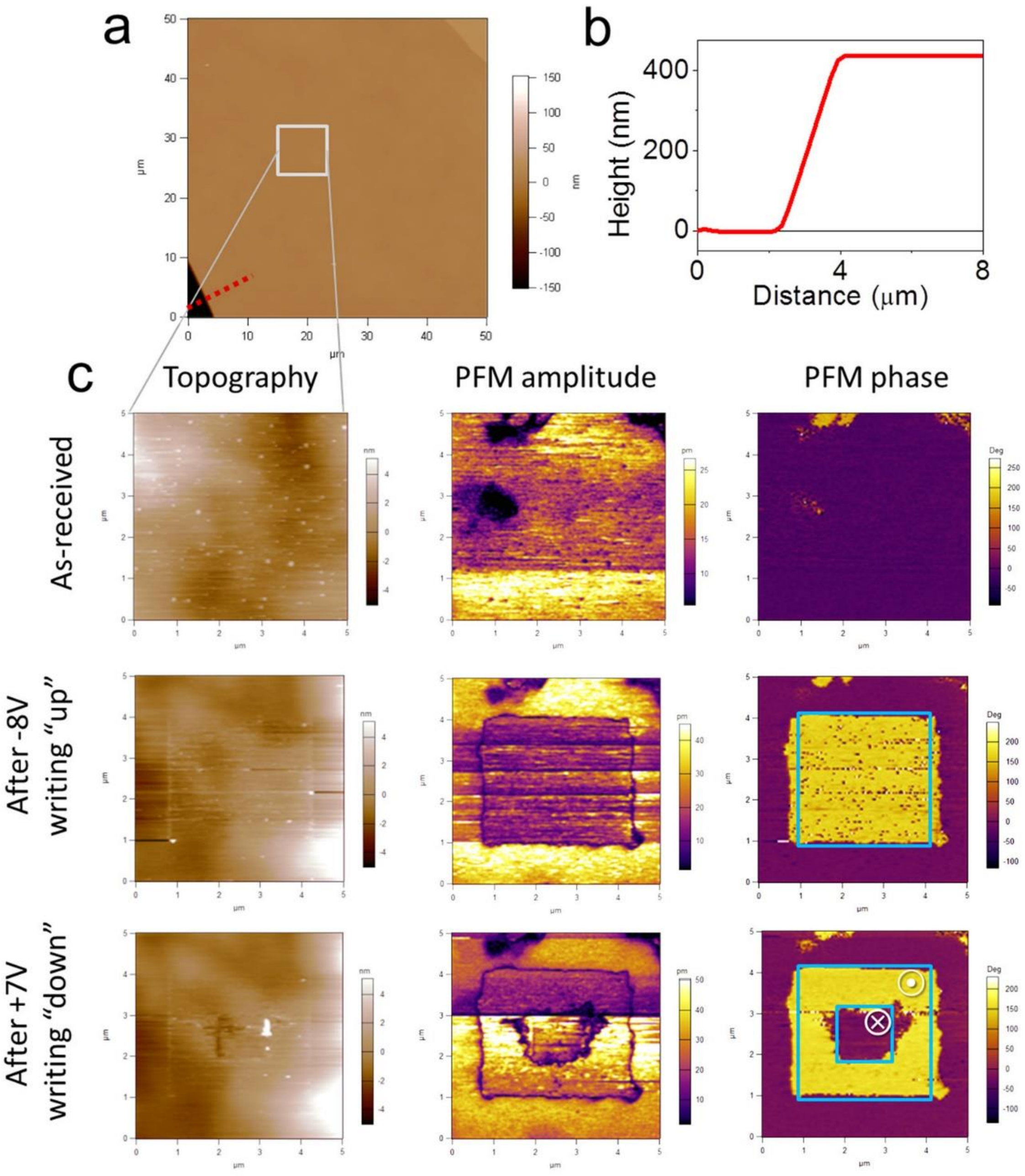

Supplementary Figure 12 | PFM measurement and polarization switching of CIPS with a thickness of 400 nm. a, The topography of the a 400-nm thick CIPS flake. b, The height profile along the dashed line in (a). c, The topography, PFM amplitude, PFM phase for the as received, after -8 V and +7 V bias writing, respectively.

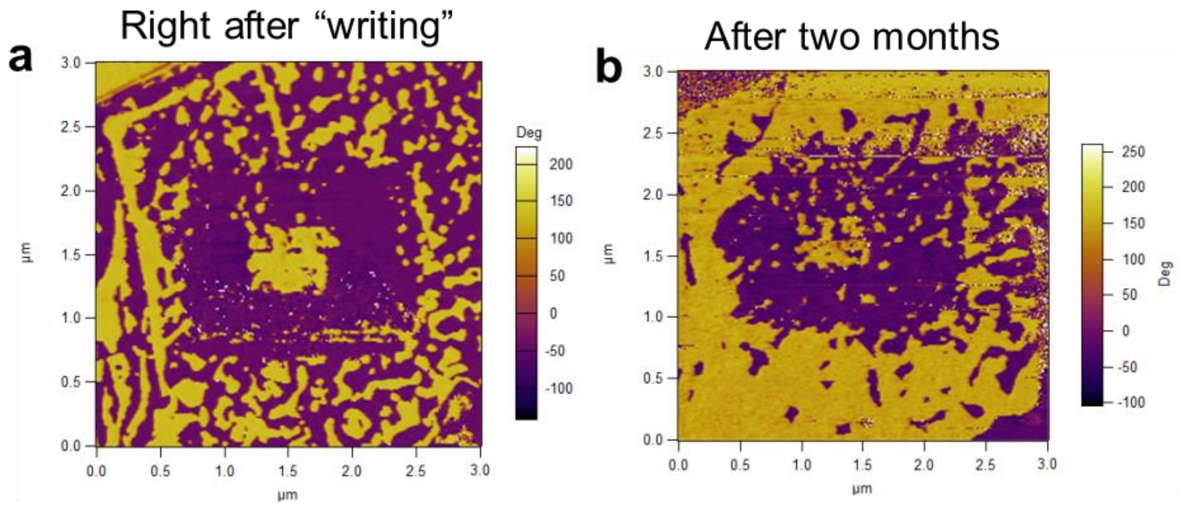

Supplementary Figure 13 | Retention performance of the switched ferroelectric domains. a-b, PFM phase image of a 30-nm-thick flake recorded (a) immediately after switching a box-in-box pattern, (b) after two months.

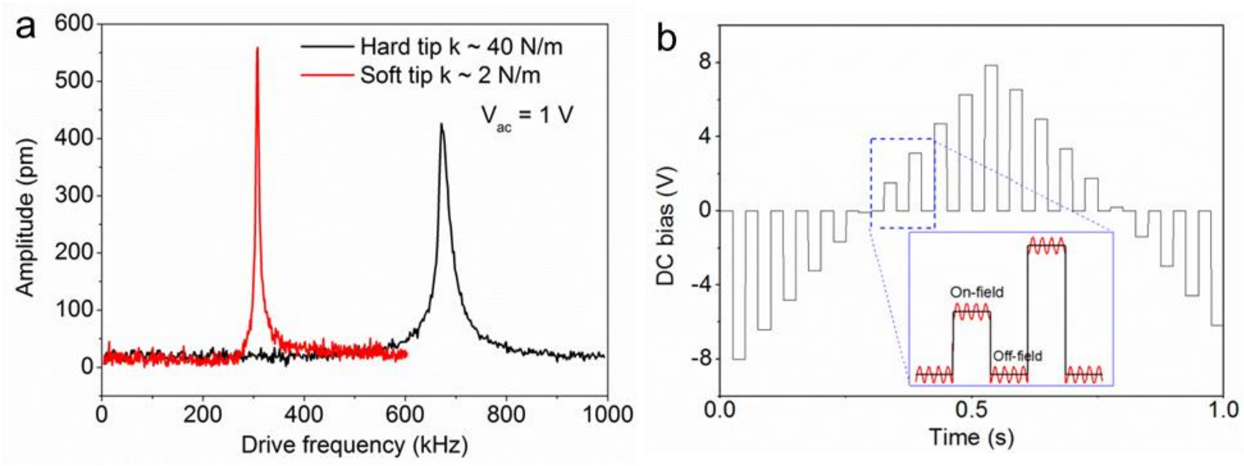

Supplementary Figure 14 | Details of the switching spectroscopy PFM. a, Typical drive-frequency-dependent PFM amplitude signal on a CIPS flake for two types of AFM tips. b, Typical voltage waveform used for polarization switching spectroscopy in resonance-enhanced PFM.

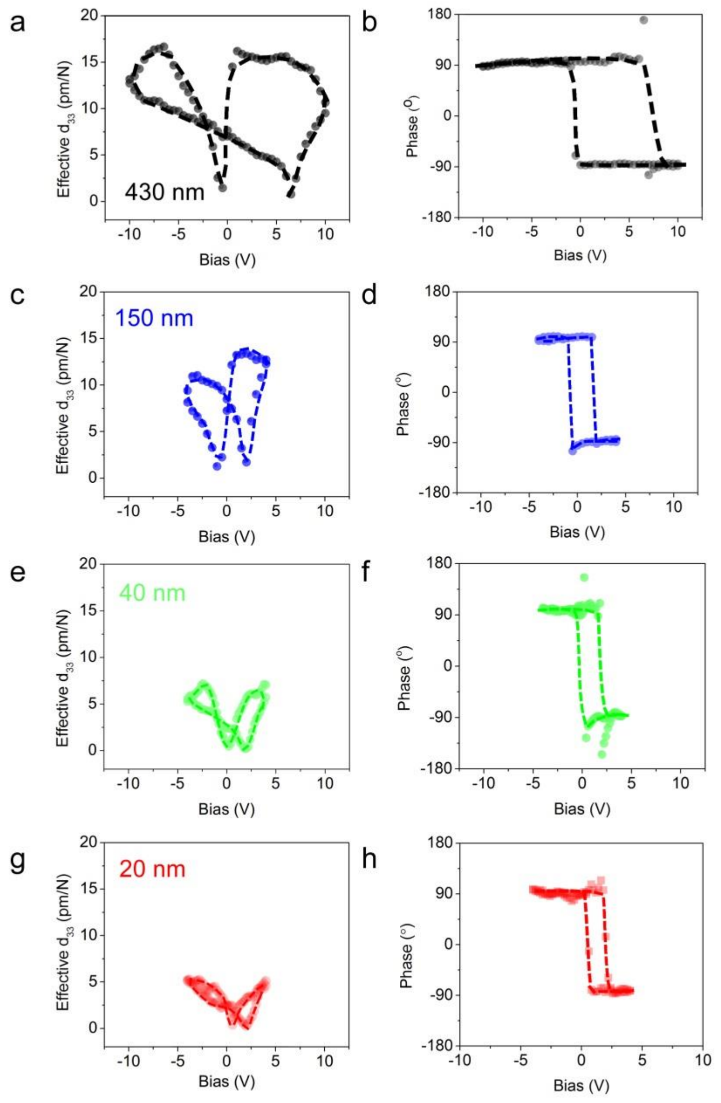

Supplementary Figure 15 | Quantitative piezoelectric hysteresis loops of CIPS flakes with different thicknesses. a-h, The effective d33 and phase hysteresis loop for the CIPS flakes with thickness of 430 nm (a,b), 150 nm (c,d), 40 nm (e,f), 20 nm (g,h).

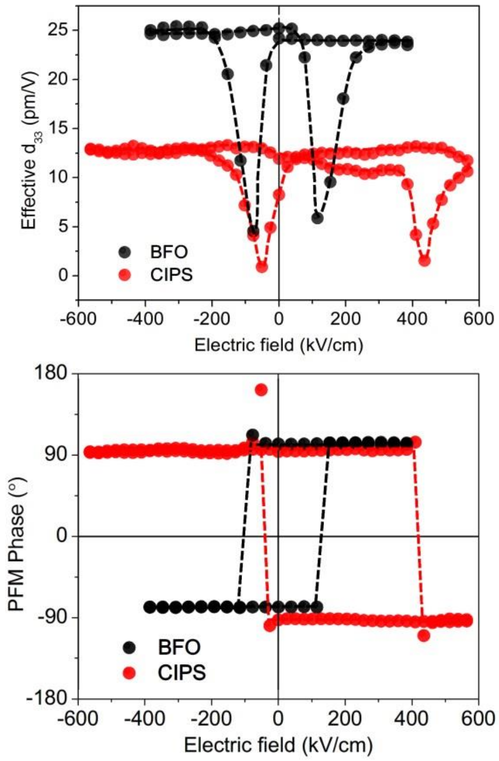

Supplementary Figure 161 | Comparison of the piezoresponse for BFO and CIPS with similar thickness of 200 nm. a, The effective d33 of a 200-nm-thick BFO thin film (black) and CIPS flake (red). b, The phase loops of a 200-nm-thick BFO thin film (black) and CIPS flake (red), the phase of CIPS is completely 180 ° out of phase with respect to BFO, indicating the negative piezoelectric coefficient of a thick CIPS flake.

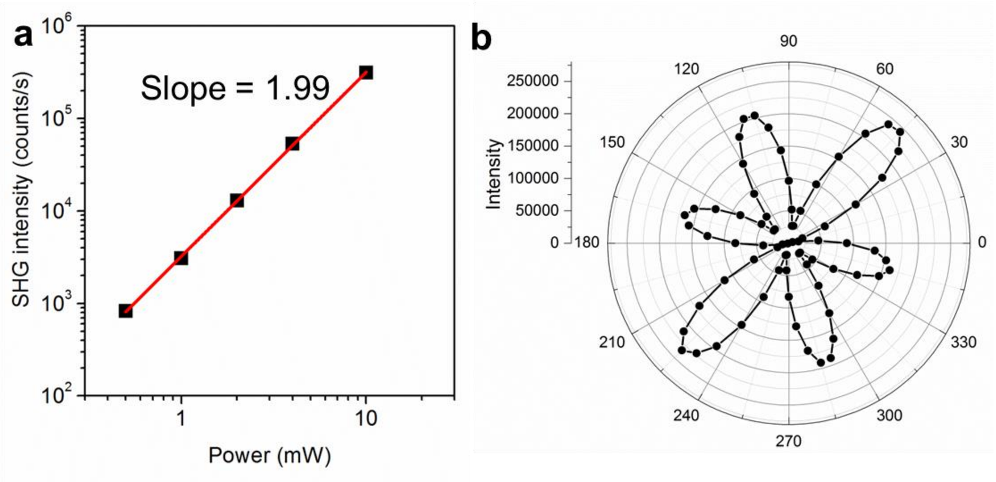

Supplementary Figure 17 | SHG characterizations of CIPS. a, Power dependence of SHG intensity, the red line shows the expected quadratic dependence. b, SHG and intensity parallel to the incident laser polarization as a function of crystal angle.

Supplementary Note 1: Ferroelectric characterization of the thick CIPS flakes.

Typical ferroelectric hysteresis loops of a 4-μm-thick CIPS flake are shown in Supplementary Fig. 1a. The inclinations of the loops are mainly due to the linear parasitic capacitance from the circuit, which can then be subtracted using the dual-pass remnant hysteresis measurement. In the dual-pass remnant hysteresis measurement, the first waveform measures both the switchable and non-switchable components of the polarization, while the second one only measures the non-switchable part. Then we can obtain the remnant-polarization hysteresis loop by subtracting the second waveform from the first one, as shown in Supplementary Fig. 1b. The remnant polarization we obtained is around 3.8 $\mu C/cm^2$, significantly larger than the previously reported value of 2.55 $\mu C/cm^2$.[1] In addition, we also observed that the remnant polarization in standard hysteresis measurement is even larger, and there is no sign of saturation with increasing electric field (Supplementary Fig. 1c). These results could be due to the competing ground states of the ferroelectricity and ferrielectricity of CIPS. In such case higher electric field can continuously drive the parallel alignment of the electric dipoles in Cu and In sublattices. In the standard hysteresis loop, there is no delay when measuring the switched polarization from one state to the other. Hence, there is no time for the parallel-aligned Cu and In dipoles to relax back. However, in the remnant hysteresis loop, this non-remnant component is removed, leaving the true remnant polarization component.

The capacitance-voltage loops under forward and reverse voltage sweeping do not coincide with each other (Supplementary Fig. 1d). Because the high ionic conductivity of the CIPS has a sizable contribution to its dielectric response at room temperature[2], this asymmetric behavior

could originate from the diode-like transport characteristic due to the asymmetric top and bottom electrical contacts (highly-doped Si versus Au). Moreover, the polarization switching is correlated with more than one bump in each branch of the hysteresis loops, suggesting a multi-step switching process, which, again, is possibly related to the competition between the stable ferrielectric order and the metastable ferroelectric order. Although the experimental observation suggests a ferrielectric ground state[1], a ferroelectric one may be stabilized by the cation disorders in the crystal[3,4].

Supplementary Note 2: Vector PFM study of the CIPS flakes to determine the polarization direction.

To verify that the polarization of CIPS is completely perpendicular to the layer plane with no in-plane component, concerted vertical (out-of-plane, OP) and lateral (in-plane, IP) PFM are carried out under off-resonance mode, which is beneficial for quantitative piezoresponse evaluation. The results are summarized in Supplementary Fig. 5 and 6. When the azimuth angle between AFM tip and the sample is 0 ° (starting reference), we observed clear piezoresponse signal and domain pattern in the OP-PFM amplitude and phase images. In contrast, IP-PFM images showed noise-level amplitude signal, and no distinguishable domain pattern could be resolved in the phase channel. Because the in-plane piezoresponse is proportional to the in-plane polarization component, perpendicular to the cantilever of the AFM tip, we rotated the sample for 90 ° to further rule out the possibility that the in-plane polarization vector happens to lie parallel to the cantilever. The OP-PFM images were well reproduced as they were not affected by the in-plane orientation of the sample. The slightly reduced amplitude signal was probably attributed to the tip wear-out. Again, no detectable signal was found in the IP-PFM images.

Finally, to rule out residual charging effect on the PFM, we have written a box-in-box pattern in the center of the scanned area and recorded down the resulting PFM images (Supplementary Fig. 5c, f, i, l). Once again, no piezoresponse signal can be detected in the IP-PFM images. These findings clearly support that the polarization vector is perpendicular to the layer plane/substrate surface.

Supplementary Note 3: DFT calculation of the ferroelectricity in bilayer CIPS.

The supercell of 40 atoms for bilayer of CIPS was used in this calculation. The structure was first fully relaxed until the force on each atom is below 0.001 Ry/Angstrom. The relaxed in-plane lattice constants were found to be 6.096 and 10.564 Angstroms, which are consistent with other reported values[5,6]. The relaxed structure, based on which the polarization is calculated, is shown in Supplementary Fig. 7. In our calculations, we find that the Cu atom is shifted 1.57 Angstrom above the middle layer of S-S layer and the In atom is shifted 0.26 Angstrom below the middle layer of S-S. This is consistent with the prior works which indicate a ferroelectric phase[1]. It is found that the phase transition between paraelectric and ferroelectric is accompanied by the hopping of Cu (upper/down, center and interlayer as clearly described by others[1,6,7]). With the relaxed supercell in hand, we first performed a self-consistent calculation and then an estimation of the polarization. The polarization is determined in the out-of-plane (z) direction to be 0.57 C/m^2. This value is much larger than the value measured by experiments, however, qualitatively it is clear that both computation and experiments show a clear ferroelectric behavior. There are several possible reasons for the discrepancy in the quantitative value of the spontaneous polarization. We approximate the original system by $CuInP_2S_6$ other than the actual chemical constitute $Cu_{0.975}InP_2S_6$[6], because it is computationally (nearly) impossibly to model the exact material system. Since the polarization is mainly contributed by the displaced Cu and In ions, the

larger polarization might be due to the high density of Cu used in the calculations. The other factor is the temperature effect since it was found that decrease of temperature may enhance the polarity of Cu and In[1]. The experiment is done at room temperature but the calculation is done at 0 K.

Supplementary Note 4: Ferroelectric switching of CIPS flakes with different thicknesses.

Supplementary Fig. 8 to 12 demonstrate the detailed evolutions of topography and correlated PFM images of CIPS flakes with thickness ranging from 4 nm to 400 nm before and after polarization switching using AFM tip on heavily-doped Si substrate. Special care was taken to ensure the applied bias was just above the coercive voltage, because higher bias would lead to the irreversible modification of the CIPS surface due to its high ionic conductivity[8]. Besides, compared with conventional oxide ferroelectrics, the written patterns in CIPS were usually worse defined. The switched domains can extend far beyond the area as defined by the AFM tip, especially in thicker films. This finding implies high domain wall energy in CIPS crystal, and similar behavior has been reported in organic molecular ferroelectric[9]. Whether it is a common characteristic in weakly-bonded ferroelectrics requires further investigations.

The ferroelectric retention behavior of the CIPS flakes was illustrated in Supplementary Fig. 13. The switched pattern was still discernible after two-month time in the 30-nm-thick CIPS flake. Surprisingly, the domain pattern in the unswitched area was greatly changed. Similar behavior was also observed in other unswitched CIPS flakes, which could be due to the internal field at the CIPS/Si interface.

Supplementary Note 5: Quantitative determination of the piezoelectric coefficient of CIPS flakes.

PFM measurements in this work were carried out both in resonant and non-resonant modes. Unless otherwise specified, the PFM imaging was usually conducted in resonance-enhanced mode using a soft tip with a spring constant of ~ 2 N/m. The local polarization switching spectroscopy in this mode employs the voltage waveform as illustrated in Supplementary Fig. 14b[10]. To eliminate the electrostatic contribution, off-field signals were used to construct the hysteresis loops shown in Fig. 3. For quantitative measurements, a very stiff tip with a spring constant of ~40 N/m was driven at 10 kHz, far away from the resonant frequency as shown in Supplementary Fig. 14a. Force curve was first performed to calibrate the inverse optical lever sensitivity (InvOLS, nm/V), which converts the photodiode deflection signal into the tip displacement[10]. The reliability of the calibration was verified using the single-domain $BiFeO_3$ capacitor, which produced an effective d_{33} about 25 pm/V, in agreement with previous report[12]. This magnitude of the piezoelectric response was further confirmed by laser scanning vibrometer[13], suggesting negligible electrostatic contribution thanks to the large stiffness of the AFM tip.

The quantitative measurements of the switching hysteresis loops showed similar behavior as those presented in Fig. 3, with larger piezoresponse observed in thicker flakes (Supplementary Fig. 15). The effective longitudinal piezoelectric coefficient d_{33} of a 200-nm-thick CIPS flake was calculated to be around 12 pm/V, about half the value of a single-domain $BiFeO_3$ (BFO) thin film with comparable thickness (Supplementary Fig. 15). Given that the dielectric permittivities of CIPS and BFO are comparable and the spontaneous polarization of BFO is at least one order of magnitude higher, the electrostrictive coefficient Q_{33} of CIPS should be much larger than BFO

since $d_{33} \sim Q_{33}P\varepsilon_r$, where P, polarization; ε_r, dielectric constant[14]. This is understandable considering the much higher elastic compliance of vdW crystals perpendicular to the layer direction compared to the in-plane one[14]. Interestingly, the phase loops of CIPS and BFO are 180 ° out-of-phase, meaning the piezoelectric coefficient of CIPS is negative (Supplementary Fig. 16). Negative piezoelectricity has been observed in hydrogen-bonded organic molecular ferroelectrics and polymer ferroelectrics such as PVDF, the common feature of which is the loose packing structure[9,16]. CIPS probably falls into the same category due to the compressibility of the vdW gap.

Supplementary Note 6: Second-harmonic generation characterization of CIPS

Optical methods such as **second-harmonic generation (**SHG) have proven to be extremely sensitive to changes in the crystal structure symmetry[17], which allows for detection of the ferrielectric to paraelectric transition as shown in Fig. 4b. Here we present a symmetry analysis showing the correlation between the ferrielectric phase and non-zero second-order susceptibility tensor components. We first investigate the excitation power dependence of the SHG in CIPS. The SHG intensity scales quadratically with excitation power as expected (Supplementary Fig. 17a), and indicates that the ultrathin samples are undamaged under the 5-10 mW of average power used in Fig. 4. In the paraelectric phase, CIPS is in the centrosymmetric crystal class 2/*m*. When the temperature decreases below T_c (~315 K), the Cu and In sublattices displace antiparallel to each other along the *z*-axis, breaking the two-fold rotational symmetry about the *y* axis. Thus, the crystal class becomes *m*, which is noncentrosymmetric. Since our excitation and collection is at normal incidence (along z axis of the crystal), the allowed second-order susceptibility tensor components simplify to χ_{yyy}, χ_{yxx}, χ_{xyx}. With these four components, we achieve a good fit to the experimental data in Fig. 4a. To gain a better intuition for the origin of

allow components, we collected the SHG intensity parallel to the incident laser polarization as a function of crystal orientation (Supplementary Fig. 17b). The six-fold pattern reflects the hexagonal symmetry of the shifted Cu and In sublattices, which are responsible for the broken inversion symmetry and ferrielectricity. The asymmetry of the lobes may indicate that there is a small strain in the sample from the exfoliation.

Supplementary References

1 Maisonneuve, V., Cajipe, V., Simon, A., Von Der Muhll, R. & Ravez, J. Ferrielectric ordering in lamellar $CuInP_2S_6$. *Phys. Rev. B* **56**, 10860-10868 (1997).

2 Maisonneuve, V. *et al.* Ionic conductivity in ferroic $CuInP_2S_6$ and $CuCrP_2S_6$. *Ferroelectrics* **196**, 257-260 (1997).

3 He, Q. *et al.* Antisite defects in layered multiferroic $CuCr_{0.9}In_{0.1}P_2S_6$. *Nanoscale* **7**, 18579-18583 (2015).

4 Ogale, S. B., Venkatesan, T. V. & Blamire, M. *Functional Metal Oxides: New Science and Novel Applications*. (John Wiley & Sons, 2013).

5 Bercha, D., Bercha, S., Glukhov, K. & Sznajder, M. Electron-Phonon Interaction as a Mechanism of Phase Transition in the $CuInP_2S_6$ Crystal. *Acta Phys. Pol. A* **126**, 1143-1145 (2014).

6 Maisonneuve, V., Evain, M., Payen, C., Cajipe, V. & Molinie, P. Room-temperature crystal structure of the layered phase $Cu^{I}In^{III}P_2S_6$. *J. Alloy Compd.* **218**, 157-164 (1995).7 Simon, A., Ravez, J., Maisonneuve, V., Payen, C. & Cajipe, V. Paraelectric-ferroelectric transition in the lamellar thiophosphate $CuInP_2S_6$. *Chem. Mater.* **6**, 1575-1580 (1994).

8 Belianinov, A. *et al.* $CuInP_2S_6$ Room Temperature Layered Ferroelectric. *Nano Lett.* **15**, 3808-3814 (2015).

9 Gao, W. *et al.* Flexible organic ferroelectric films with a large piezoelectric response. *NPG Asia Mater.* **7**, e189 (2015).

10 Jesse, S. *et al.* Direct imaging of the spatial and energy distribution of nucleation centres in ferroelectric materials. *Nat. Mater.* **7**, 209-215 (2008).

11 Proksch, R. In-situ piezoresponse force microscopy cantilever mode shape profiling. *J. Appl. Phys.* **118**, 072011 (2015).

12 Chu, Y. H. *et al.* Domain Control in Multiferroic $BiFeO_3$ through Substrate Vicinality. *Adv. Mater*. **19**, 2662-2666 (2007).

13 Kui, Y. & Eng Hock Tay, F. Measurement of longitudinal piezoelectric coefficient of thin films by a laser-scanning vibrometer. *IEEE Trans. Ultrason. Ferroelectr. Freq. Control* **50**, 113-116 (2003).

14 Devonshire, A. F. CIX. Theory of barium titanate—Part II. *Lond.Edinb.Dubl.Phil.Mag.* **42**, 1065-1079 (1951).

15 Gao, Y. *et al.* Elastic coupling between layers in two-dimensional materials. *Nat. Mater.* **14**, 714-720, (2015).
16 Bystrov, V. S. *et al.* Molecular modeling of the piezoelectric effect in the ferroelectric polymer poly (vinylidene fluoride)(PVDF). *J. Mol. Model.* **19**, 3591-3602 (2013).
17 Shen, Y.-R. The Principles of nonlinear optics (Wiley, 1984).
